# Radiomics for liver tumours

**DOI:** 10.1007/s00066-020-01615-x

**Published:** 2020-04-15

**Authors:** Constantin Dreher, Philipp Linde, Judit Boda-Heggemann, Bettina Baessler

**Affiliations:** 1 Department of Radiation Oncology, University Hospital Mannheim, Medical Faculty of Mannheim, University of Heidelberg, Theodor-Kutzer Ufer 1–3, 68167 Mannheim, Germany; 2 Department of Radiation Oncology, Medical Faculty and University Hospital Cologne, University of Cologne, Kerpener Str. 62, 50937 Cologne, Germany; 3 Institute of Diagnostic and Interventional Radiology, University Hospital Zurich, Raemistrasse 100, 8091 Zurich, Switzerland

**Keywords:** Artificial intelligence, Big data, Magnetic resonance imaging, Computed tomography, Stereotactic body radiation therapy

## Abstract

Current research, especially in oncology, increasingly focuses on the integration of quantitative, multiparametric and functional imaging data. In this fast-growing field of research, radiomics may allow for a more sophisticated analysis of imaging data, far beyond the qualitative evaluation of visible tissue changes. Through use of quantitative imaging data, more tailored and tumour-specific diagnostic work-up and individualized treatment concepts may be applied for oncologic patients in the future. This is of special importance in cross-sectional disciplines such as radiology and radiation oncology, with already high and still further increasing use of imaging data in daily clinical practice. Liver targets are generally treated with stereotactic body radiotherapy (SBRT), allowing for local dose escalation while preserving surrounding normal tissue. With the introduction of online target surveillance with implanted markers, 3D-ultrasound on conventional linacs and hybrid magnetic resonance imaging (MRI)-linear accelerators, individualized adaptive radiotherapy is heading towards realization. The use of big data such as radiomics and the integration of artificial intelligence techniques have the potential to further improve image-based treatment planning and structured follow-up, with outcome/toxicity prediction and immediate detection of (oligo)progression. The scope of current research in this innovative field is to identify and critically discuss possible application forms of radiomics, which is why this review tries to summarize current knowledge about interdisciplinary integration of radiomics in oncologic patients, with a focus on investigations of radiotherapy in patients with liver cancer or oligometastases including multiparametric, quantitative data into (radio)-oncologic workflow from disease diagnosis, treatment planning, delivery and patient follow-up.

## Introduction

With the introduction of radiomics, both oncologic radiology and radiation oncology have gained a highly promising tool for more sophisticated quantitative tumour analysis. Current research, especially in oncology, increasingly focuses on the integration of quantitative, multiparametric and functional imaging data. In this fast-growing field of research, radiomics may allow for an all-encompassing analysis of quantitative imaging data, far beyond the qualitative evaluation of visible tissue changes. Through use of multiparametric, quantitative imaging data, a more tailored and tumour-specific diagnostic work-up and individualized treatment concepts may be applied for oncologic patients in the future.

The scope of current research in this innovative field is to identify and critically discuss possible application forms of radiomics, which is why this review tries to summarize current knowledge about interdisciplinary integration of radiomics in oncologic patients. This review specifically focusses on investigations on radiotherapy in patients with liver cancer, including the (radio)-oncologic workflow from disease diagnosis, treatment planning, delivery and patient follow-up.

Radiomics includes not only the commonly known quantitative data features derived from the pixel grey-level histogram, i.e. mean, maximum, minimum and median parameters, but also the analysis of imaging data based on computerized mathematical and statistical feature extraction, describing further quantitative characteristics of the segmented regions with regard to tissue heterogeneity, compacity etc. [1]. Radiomic analysis of quantitative imaging parameters may further characterize both tumour and normal tissue and even predict tumour response and toxicity by incorporating these data into statistical or advanced machine learning models [2–5].

There are already some promising data about radiomics clarifying mammographic findings suspicious for cancer [6] or predicting mutational status in glioblastomas [7]. Additionally, there are still few but promising data regarding the use of radiomics in oncologic liver imaging [8]. Morphological and functional characterization of liver tumours with and without contrast-enhanced sequences is the state of the art in oncologic liver imaging. Recent radiomics studies demonstrated for the first time the predictive value for different liver tumours, such as the grade of hepatocellular carcinoma (HCC) or the differential diagnosis of other primary or secondary liver tumours and benign liver lesions [9–13]. Table 1 summarizes these studies.

**Table 1:** Radiomics for predictive use

Author	Aims	Imaging modality	Number (training and validation sets, where available)	Conclusion
Lewis et al. [9]	To distinguish hepatocellular carcinoma (HCC) from other primary liver cancers (intrahepatic cholangiocarcinoma [ICC] and combined HCC-ICC) through volumetric quantitative apparent diffusion coefficient (ADC) histogram parameters and LI-RADS categorization	MRI	63	Combination of quantitative ADC histogram parameters and LI-RADS categorization yielded the best prediction accuracy for distinction of HCC compared to ICC and combined HCC-ICC
Wu et al. [10]	To evaluate the feasibility of using radiomics with precontrast MRI for classifying HCC and hepatic haemangioma (HH)	MRI	369	Radiomics-based assessments could be used to distinguish between HCC and HH on precontrast images, thereby allowing noninvasively efficient identification and minimizing errors from visual inspection
Oyama et al. [11]	To evaluate the accuracy for classification of hepatic tumours	MRI	37 HCCs, 23 metastatic tumours, and 33 HHs	Using texture analysis or topological data analysis allows for classification of the three hepatic tumours with considerable accuracy
Wu et al. [12]	To predict histopathological grading for HCC cases	MRI	170	A computed radiomics signature itself or combined with clinical factors could help to classify the patients into high-grade or low-grade HCC

The columns *Aims* and *Conclusion* are directly based on the original work as cited in the column *Author* (wording partly adapted).

*CECT* contrast-enhanced computed tomography, *ER* early recurrence, *HCC* hepatocellular carcinoma, *LI-RADS* Liver Imaging Reporting and Data System, *MRI* magnetic resonance imaging, *MVI* microvascular invasion

The present review article aims at summarizing the work which has been done in the field of radiomics in liver imaging until today, with a special focus on relevant topics from the field of radiation therapy. Firstly, it will summarize the current indications for radiotherapy in the liver, before summarizing the current literature covering radiomics for treatment planning in the liver. A short section on radiomics for monitoring and follow-up will then be followed by a summary of radiomics in the imaging of the post-treatment liver. Finally, we will give a short overview about current limitations in the field of radiomics.

## Indications for radiotherapy

With the introduction of image guidance and conformal radiotherapy techniques, the treatment of both primary and secondary liver tumours has experienced significant improvement over the past few years, leading to increased local control rates and decreased normal tissue toxicity [14–19].

Recent clinical data indicate that additional local therapy to each metastatic lesion can prolong the overall survival of oligometastatic patients [20–22]. Furthermore, immunotherapy enables new treatment options for several tumour entities, especially in combination with radiotherapy [23].

In patients with oligometastatic disease, especially hepatic metastases exhibit different treatment courses. Patients with up to five lesions are increasingly treated with aggressive metastasis-directed treatment options, improving survival in some patients, even in case of recurrent liver metastases [24, 25]. In comparison to other locally ablative treatment options, resection is anticipated for patients with isolated liver metastases, although being characterized with an increased post-procedure morbidity [24, 26].

Consequently, minimally invasive options like transarterial chemoembolization (TACE) and radiofrequency/microwave ablation (RFA/MWA) have been evaluated, demonstrating good high local control rates and safety records [27–30]. SBRT as a completely non-invasive procedure is an evolving alternative, showing similar or even better clinical outcomes [18, 31]. Unfortunately, to date, prospective data about the different local ablative treatment options are lacking. Nevertheless, ongoing technical improvements provide promising data, especially in the case of SBRT of liver metastases, with median overall survival rates of 31.5 months in colorectal cancer patients [32].

Primary liver cancers are generally intended to be resected. However, in case of inoperability, prospective studies on SBRT and SBRT with optional TACE in primary HCC showed promising 18-month and 3‑year overall survival rates of 72% and up to 67%, respectively [33, 34]. SBRT is even feasible in patients with advanced-stage HCC, with 3‑year overall survival (OS) rates of 24.3% and 3‑year local control rates of 78.1% [35]. Furthermore, there is increasing evidence that SBRT may even be superior to TACE regarding survival and recurrence, and especially after prior TAE/TACE treatment [36, 37]. In case of SBRT in cholangiocellular carcinoma (CCC) patients, 3‑year OS rates are about 21%, with increasing local control rates depending on delivered dose (biological effective radiation dose [max] >91 Gy [α/β = 10 Gy]) [38].

Although RFA is regarded as the main alternative treatment option in unresectable HCC, retrospective data indicate the possible superiority of SBRT as compared to RFA with regard to tumours >2 cm [18]. With competing data being published for the comparison of SBRT and RFA, prospective trials are needed [39]. If transplantation is indicated, a combination of neoadjuvant SBRT and TACE provides promising remission and reasonable overall survival rates [40, 41].

## Radiomics for treatment planning

### Target volume definition: automatic segmentation of target volumes and organs at risk

The functional capability of the liver to regenerate and proliferate has been used for a long time in liver surgery [42]. When it comes to a healthy liver, 80% of the organ can be removed. Although the whole liver exhibits a low radiation tolerance, potentially leading to the serious condition of radiotherapy-induced liver disease (RILD) [43–49], the regenerative potential and the parallel radiobiological character of the liver allows for application of high doses to a defined volume without compromising liver function [50].

Patients with liver metastases usually have a functionally “healthy” liver. Previous oncological treatments like chemotherapy or immunotherapy can influence liver function [51, 52]. How these previous therapies influence the individual radiation tolerance is still unknown and subject to research. In contrast to liver metastases, most patients with primary liver tumours have liver cirrhosis, which limits local ablative and surgical treatments due to the subsequently impaired liver function [53, 54]. Current understanding of the radiation-induced impairment of liver function sees hepatic veno-occlusive disease as the pathological hallmark of liver injury [55], while at the same time, the vulnerability of (hepato)biliary structures has to be taken into account, especially for centrally located liver cancer [56, 57].

Tolerance doses/dose constraints for therapy planning in organs at risk have been investigated for decades, which is why tolerance doses also rely on data being gained with different radiotherapy techniques and—most importantly—with irradiated volumes significantly larger than the volumes in modern radiotherapy techniques such as SBRT [58]. Consequently, organs at risk (OAR) constraints have to be re-evaluated in order to allow for more tailored treatment concepts and monitoring during treatment on the basis of clinical and multiparametric quantitative imaging data. With SBRT being characterized by different biological efficacy as compared to standard radiotherapy, dose escalation may be performed under consideration of dose constraints based on analyses on clinical toxicity [59–63].

**Fig. 1:**
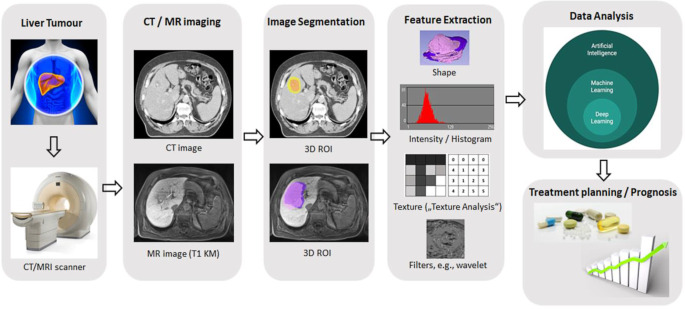
Exemplary radiomics workflow for liver imaging. Schematic illustration of the entire patient journey including image acquisition, analysis utilizing radiomics, and the derived patient-specific therapy and prognosis. Symptomatic patients undergo CT (computed tomography) or MR (magnetic resonance) scans. After image segmentation, radiomic features are extracted. High-level statistical modelling involving machine learning is applied for disease classification, patient clustering and individual risk stratification

Radiomic analysis and its use during treatment planning might further contribute to improved dose escalation schemes and allow for future automation and increased robustness of target volume delineation. In addition, a more reliable identification of high-risk regions with possible tumour infiltration (clinical target volume [CTV]) might be enabled by radiomics, since radiomics extends beyond the visible tumour infiltration and provides quantitative data on potential tumour extension [64, 65]. An exemplified workflow for the extraction of radiomic features is shown in Fig. 1.

Radiomic features and clinical parameters have been combined in a radiomics signature to preoperatively estimate early recurrence in patients with HCC [66, 67]. In addition, several promising studies (Table 2) have been conducted in liver tumours, where microscopic characteristics could be identified based on radiomics, thus potentially enabling the detection of microscopic tumour infiltration of healthy liver tissue with the inherent potential of more precise clinical target volume delineation in the future. Furthermore, evidence has been provided about prediction of microvascular invasion of HCC using radiomics on contrast-enhanced CT [68–70]. Using a radiomics approach based on contrast-enhanced T1-weighted MRI in the hepatobiliary phase, T‑cell infiltration in the tumour and peritumoural margin could be quantified [71]. Ex-vivo investigations in mice demonstrated the detection of microscopic tumour infiltration locations by the radiomic histogram feature skewness in liver single-photon emission CT (SPECT) imaging [72]. Thus, further specification of regions with a high risk of liver tumour infiltration may be enabled by radiomics in the future [70, 71].

**Table 2:** Auto-planning and predictive use of radiomics

Author	Aims	Imaging modality	Number, (training (T) and validation (V) set, where available)	Conclusion
Chen et al. [71]	To develop a radiomics model based on gadolinium-ethoxybenzyl-diethylenetriamine (Gd-EOB-DTPA)-enhanced MRI for pretreatment prediction of immunoscore in HCC	MRI	207 T: 150 V: 57	MRI-based combined radiomics nomogram shows effectiveness in predicting immunoscore in HCC
Shan et al. [66]	To predict recurrence of HCC (hepatocellular carcinoma) after curative treatment	CECT	156 T: 109 V: 47	A radiomics model effectively predicts early recurrence (ER) of HCC and is more efficient than conventional imaging features and models
Xu et al. [68]	To predict microvascular invasion (MVI) and clinical outcomes in patients with HCC	CECT	495 T: 350 V: 145	The computational approach demonstrates good performance for predicting MVI and clinical outcomes
Vivanti et al. [88]	To automatically delineate liver tumours in longitudinal CT studies	CECT	31	The system showed the ability to predict failures and the ability to correct them
Vorontsov et al. [89]	To bring up a semi-automatic tumour segmentation method	CECT	40	The proposed method can deal with highly variable data
Bakr et al. [69]	To predict MVI	CECT	28	RF (Radiomic features) computed with single-phased or combined-phased images were correlated with MVI
Peng et al. [70]	To develop and validate a radiomics nomogram for the preoperative prediction of prognosis in patients with HCC undergoing partial hepatectomy	CECT	304 T: 184 V: 120	Radiomics nomogram showed excellent performance for the individualized and non-invasive estimation of disease-free survival, which may help clinicians better identify patients with HBV-related HCC who can benefit from the surgery
Zhou et al. [67]	To predict ER of HCC	CECT	215	Radiomics signature was a significant predictor for ER in HCC
Liu et al. [86]	To develop and validate a learning-based method to derive electron density from routine anatomical MRI for potential MRI-based SBRT treatment planning; CT and MRI for CT synthesis	(co-registered) CT and MRI	21	Image similarity and dosimetric agreement between synthetic CT and original CT
Fu et al. [90]	To expedite the contouring process for MRI-guided adaptive radiotherapy (MR-IGART), a convolutional neural network deep-learning model is proposed to accurately segment the liver, kidneys, stomach, bowel and duodenum in 3D MR images	CEMRI	120 T: 100 V: 10 Test: 10	The proposed method can automatically segment the liver, kidneys, stomach, bowel, and duodenum in 3D MR images with good accuracy
Zhang et al. [91]	To build a knowledge-based model of liver cancer for auto-planning	CECT	70 T: 20	Auto-planning shows availability and effectiveness
Li et al. [65]	CT textural feature analysis for the stratification of single large HCCs >5 cm, and the subsequent determination of patient suitability for liver resection (LR) or transcatheter arterial chemoembolization (TACE)	CECT	130	Texture analysis demonstrated the feasibility of using HCC patient stratification for determining the suitability of LR vs. TACE

The columns *Aims* and *Conclusion* directly based on the original work as cited in the column *Author* (wording partly adapted).

*CECT* contrast-enhanced computed tomography, *ER* early recurrence, *HCC* hepatocellular carcinoma, *MRI* magnetic resonance imaging, *MVI* microvascular invasion

Independently of current research on standardization of dose prescription, target volume definition is increasingly based on functional imaging, such as metabolic imaging with positron-emission tomography (PET) and functional sequences of MRI, including diffusion-weighted imaging (DWI) [73, 74]. PET-imaging allows for quantitative evaluation of metabolic function in the liver tumour and normal liver tissue, thus leading to the possibility of adapted target delineation and dose reduction in the normal liver tissue [75]. In contrast to PET-based image-guided treatment planning, contrast-enhanced MRI (including with liver-specific agents) is already part of daily clinical practice in treatment planning for patients with liver cancer [76].

Contrast-enhanced T1-weighted sequences allow for morphological target delineation, but functional imaging sequences such as DWI provide additional information about perfusion fractions by intravoxel incoherent motion (IVIM), cellularity by the ADC and even tissue complexity by kurtosis evaluation [77–84]. Consequently, research is conducted on incorporation of functional MRI sequences into the daily clinical practice of treatment planning of upper abdominal tumours [85]. Besides this, Liu et al. showed that synthetic CT datasets can be generated from MRI to ensure accurate liver SBRT [86].

Adding to radiomics, the increasingly used deep learning methods are able to learn directly from the data, thus circumventing the need for handcrafting of discriminative imaging features representing the key concept behind radiomics. Recently, automatic CTV segmentation has been shown by using convolutional neural networks (CNN), which appear to be especially useful for image segmentation tasks [87–89]. Deep learning-based auto-contouring of the tumour volume has been shown to be at least as efficient as manual contouring of the OAR for MRI-guided adaptive radiotherapy [90, 91]. Further promising performance for deep learning-based automatic segmentation approaches of the macroscopic gross tumour volume can be found in the ongoing “Liver Tumour Segmentation Challenge (LiTS)” (https://competitions.codalab.org/competitions/17094).

Independent of the images to be integrated into treatment planning, robust image registration (deformable or rigid registration) has to be performed to adjust for differences in image acquisition and organ movement, thus allowing for topographically correct target delineation [92].

### Adaptive radiotherapy: dose painting

The idea of adaptive radiotherapy summarizes the goals of patient-specific and tumour-tailored treatment concepts, allowing for both adapted treatment planning with locally volitional dose escalation in malignancies and reduction of dose exposure in the surrounding normal tissue. This is already possible due to the highly conformal dose application in modern treatment techniques such as image-guided radiation therapy (IGRT) and SBRT. However, daily image guidance with cone beam computed tomography (CBCT) scans and consecutive plan adaptions request a high cost in resources while only providing low-resolution CBCT images. The latest introduction of hybrid MR-guided radiotherapy gadgets might finally allow for online imaging and plan adaption based on high-resolution images, especially with respect to the soft tissue of upper abdominal organs [93]. Furthermore, this also allows for taking into account tumour heterogeneity based on quantitative, functional imaging data, exceeding the purely morphological characteristics and potentially allowing for earlier evaluation of tumour response and local control failure already at the beginning of radiotherapy and in the meantime (Table 3; [91, 94]).

**Table 3:** Radiomics for predicting patient outcome

Author	Aims	Imaging modality	Number, (training (T) and validation (V) set, where available)	Conclusion
Cai et al. [99]	To develop and validate a radiomics-based nomogram for the preoperative prediction of posthepatectomy liver failure (PHLF) in patients with HCC	CECT	112 T: 80 V: 32	A nomogram based on the Radiomics-score, model for end-stage liver disease (MELD), and performance status (PS) can predict PHLF
Ibragimov et al. [100]	To predict toxicity beyond the existing dose/volume histograms	CECT	125	A framework offers clinically accurate tools for hepatobiliary toxicity prediction and automatic identification of anatomical regions that are critical to spare during stereotactic body radiation therapy
Park et al. [101]	To develop and validate a radiomics-based model for staging liver fibrosis	Gadoxetic acid-enhanced hepatobiliary phase MRI	436	Radiomics analysis of gadoxetic acid-enhanced hepatobiliary phase images allows for accurate diagnosis of liver fibrosis
Dogan et al. [94]	To determine the changes in image texture features (delta-radiomics) measured on daily low-field MRI and whether delta-radiomics features could be used to assess treatment response and predict patient outcomes	MRI	10	Dogan et al. demonstrated that three delta-radiomics texture features extracted from low-field MRI during SBRT in liver were able to differentiate between local disease control and local control failure

The columns *Aims* and *Conclusion* are directly based on the original work as cited in the column *Author* (wording partly adapted).

*CECT* contrast-enhanced computed tomography, *ER* early recurrence, *HCC* hepatocellular carcinoma, *MRI* magnetic resonance imaging, *MVI* microvascular invasion

Independent of general outcome analysis, the incorporation of radiomics into treatment planning may further improve the analysis and prediction of normal tissue toxicity, as proposed by the QUANTEC group (Quantitative Analysis of Normal Tissue Effects in the Clinic) [95, 96]. There are already promising data with regard to toxicity after radiotherapy of head and neck and lung cancers [97, 98]. However, regarding toxicity after treatment of liver tumours, there is still little data available and future investigations are needed. Cai et al. demonstrated the prediction of liver failure after hepatectomy in patients with HCC by preoperative radiomics-based nomograms [99]. A predictive nomogram and a CNN including imaging data with high performance for toxicity prediction after liver SBRT are already available [57, 100]. Generally, toxicity analysis in healthy liver tissue should be improved even more, since MRI enables accurate liver function analysis and radiomics analysis allows for accurate staging of liver fibrosis and may prevent and, vice versa, predict RILD [101–104].

## Monitoring/follow-up

First promising results regarding the predictive potential of radiomics for local response after radiotherapy and TACE have recently been demonstrated [105–107] and are listed in Table 4. Treatment response of liver metastases after TACE has been determined using a radiomics-based analysis resulting in area under the curve (AUC) in receiver operating characteristics (ROC) of up to 0.83 [105]. Radiomic features have been integrated into multivariate models predicting local control and overall survival rates after radiotherapy of HCC with an AUC of 0.80 [106]. Furthermore, by combining radiomics features with clinical data, survival prediction might even be improved in patients with HCC [13, 107, 108]. Even in case of cholangiocarcinoma, pre-operative MRI was able to predict early recurrence, especially in combination with immunohistochemical markers [109].

**Table 4:** Radiomics for monitoring/follow-up

Author	Aims	Imaging modality	Number, (training (T) and validation (V) set, where available)	Conclusion
Reimer et al. [105]	To determine whether post-treatment MRI-based texture analysis of liver metastases may be suitable for predicting therapy response to transarterial radioembolization (TARE) during follow-up	CEMRI	37	The model indicates the potential of MRI-based texture analysis at arterial- and venous-phase MRI for the early prediction of progressive disease after TARE
Cozzi et al. [106]	To predict overall survival and local control	Non-contrast CT	138	Survival could be predicted using a radiomics signature made by a single shape-based feature
Kim et al. [107]	To predict survival (overall and progression-free survival)	CECT	88	A combination of clinical and radiomic features better predicted survival
Mokrane et al. [108]	To enhance clinicians’ decision-making by diagnosing HCC in cirrhotic patients with indeterminate liver nodules using quantitative imaging features	CECT	178 T: 142 V: 36	Radiomics can be used to non-invasively diagnose HCC in cirrhotic patients with indeterminate liver nodules, which could be used to optimize patient management
Donghui et al. [13]	To identify aggressive behaviour and predict recurrence of HCC after liver transplantation (LT)	CECT	133 T: 93 V: 40	Radiomics signature extracted from CT images may be a potential imaging biomarker for liver cancer invasion and enable accurate prediction of HCC recurrence after LT
Zhao et al. [109]	To investigate the combined predictive performance of qualitative and quantitative MRI features and prognostic immunohistochemical markers for the ER of intrahepatic mass-forming cholangiocarcinoma (IMCC)	CEMRI	47	The combined model was the superior predictive model of ER

The columns *Aims* and *Conclusion* are directly based on the original work as cited in the column *Author* (wording partly adapted).

*CECT* contrast-enhanced computed tomography, *ER* early recurrence, *HCC* hepatocellular carcinoma, *MRI* magnetic resonance imaging, *MVI* microvascular invasion

## Imaging of the post-treatment liver

**Fig. 2:**
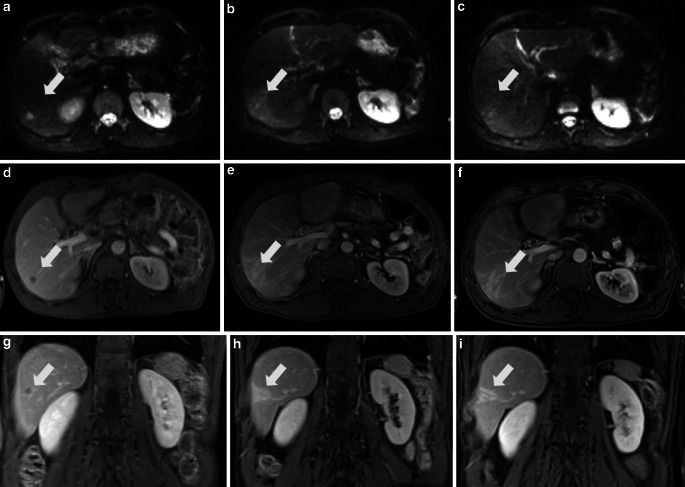
Longitudinal changes of a hepatic metastasis in the right liver lobe after stereotactic radiotherapy (SBRT). MRI sequences: diffusion-weighted imaging (DWI) transverse (**a–c**), contrast-enhanced T1-weighted sequence (portal-venous phase) transverse (**d–f**) and coronal (**g–i**). MRI prior to SBRT (**a,** **d,** **g**), 3 months after SBRT (**b,** **e,** **h**) and 12 months after SBRT (**c,** **f,** **i**). Morphological response of DWI restriction, T1‑w hypointensity after SBRT with longitudinal reduction of peritumoral changes of the normal tissue. *White arrows* highlight the region of interest including the hepatic metastasis in the right liver lobe and the peritumoral changes after SBRT

In addition to a temporary/reversible decline in metabolic function, liver tissue is characterized by distinct macroscopic, microscopic and CT/MR-morphological changes after radiotherapy; exemplary changes are given in Fig. 2. In patients with sufficient baseline liver function prior to radiotherapy, a compensatory hypertrophy of the untreated liver may occur after radiotherapy [110]. Repetitive imaging during and after radiotherapy demonstrated that changes in metabolic liver function are also accompanied by distinct changes in quantitative imaging data of the liver tumour and the normal liver tissue [53, 111–114].

Nevertheless, there are still few data about quantitative imaging encompassing the whole treatment course of liver tumours, especially with respect to normal tissue alterations. Multiparametric imaging might allow for both specification in staging examinations and acceleration by the use of quantitative imaging parameters, potentially replacing extensive amounts of qualitative imaging sequences for visible, mainly qualitative evaluation of the tumour and normal tissue. Sequences such as DWI with quantitative ADC maps are characterized by these possibilities and allow for functional analysis of the tumour response after SBRT [115].

As a consequence, integration of this quantitative data might lead to an improvement of oncologic patient management in future, especially with respect to the large amount of radiological data in image-guided radiation oncology. Investigations in this field emphasize the role of big data in oncology. With regard to radiation oncology, the so-called radiomics concept with computerized algorithm-based parameters may be successfully integrated into daily clinical practice, supporting decision-making and improving the workflow of radiation therapy.

## Current limitations of radiomics

As reviewed above, radiomics for radiotherapy of liver tumours is highly promising, but we are still in need of further data about its validity and optimal usage for a reliable translation to daily clinical practice. The correct and robust application of radiomics analysis has to be investigated, since the algorithm-based analysis of quantitative date is not standardized within different institutions, or even within single institutions, and can easily be performed differently. Independently of software- and hardware-induced variability [116–121], texture analysis is also hindered by uncertainties in patient immobilization and organ movements, especially with regard to MRI examinations [122, 123]. On top of this, its usage for treatment decisions and treatment planning has to be investigated in prospective trials including ex-vivo, in-vivo volunteer and in-vivo patient examinations allowing for founded conclusions about its usage. Furthermore, the analysis of radiomics necessitates sufficient informational technique (IT) infrastructure with high data storage capacity and computational performance in image analysis, which is why the introduction of radiomics analysis in radiation oncology requests an IT structure similar to the one in radiology institutions.

Independent of providing sufficient software and hardware, incorporation of radiomics into radiation oncology also necessitates interdisciplinary teams, including medical doctors (clinical radiation oncologists, clinical radiologists), medical physicists and most importantly, computer scientists. The evaluation of radiomics on the basis of incorporating the imaging data together with clinical and histological data into artificial intelligence techniques, such as deep convolutional neural networks, is of utmost importance.

## Conclusion

Due to the introduction of modern radiation treatment techniques such as SBRT and IGRT, radiotherapy is capable of successfully treating both primary and secondary liver tumours, with promising local control rates. With the possibilities of multiparametric, quantitative data, including the deeper radiomics analysis, information exceeding qualitative evaluation of visible changes may be included into oncologic radiology and radiation oncology. Hybrid MR-guided radiotherapy gadgets may summarize these techniques and, together with further evaluations with artificial intelligence, patient-specific and tumour-tailored radiation treatment may become a reality.

As a consequence, prospective multi-institutional trials for liver radiotherapy are needed, with standardized image acquisition integrating radiomics quality scores to improve the research quality and to increase the influence of radiomics, further analysing radiomics’ impact in patients with liver tumours and evaluating the true potential of the predictive models.
